# Supplementary material for: Content-rich biological network constructed by mining PubMed abstracts
Source: BMC Bioinformatics. 2004 Oct 8;5:147. doi: 10.1186/1471-2105-5-147 (PMC528731; doi:10.1186/1471-2105-5-147)
Supplement: Additional File 2 — The original results of the above study (non-essential files are deleted to keep the file size under the limit set by BMC bioinformatics). [file 1471-2105-5-147-S2.bz2 › chilibotAdditionalFile2/dip50/327ID9546397E3626/html/TFRC_TF.html]

 


 **TFRC** and **TF** 
  
Found 3207 abstracts in PubMed, retrieved 50.  
 

 What does Google say? 
 PDF only 
| .edu only 

---

**Interactive relationship** (e.g. stimulation, inhibition, etc)

**Stimulatory relationship**- Transferrin  [ **TF** ]  receptor  [ **TFRC** ]  TfR  [ **TFRC** ]  facilitates cellular iron uptake by mediating endocytosis of its ligand, iron loaded transferrin  [ **TF** ] .  Ref: 12881306 BloodBlood,
**Neutral relationship**- Recent studies have revealed that the wild type hemochromatosis protein HFE interacts with the transferrin  [ **TF** ]  receptor  [ **TFRC** ]  TfR  [ **TFRC** ]  and modulates TfR  [ **TFRC** ]  mediated iron uptake by cells.  Ref: 12791823 J Nucl Med, 2003
- Coimmunoprecipitation studies using solubilized cell extracts demonstrated that transferrin  [ **TF** ]   **Tf**  competed with HFE for binding to the transferrin  [ **TF** ]  receptor  [ **TFRC** ]  TfR  [ **TFRC** ]  similar to previous in vitro studies using soluble truncated forms of HFE and the TfR  [ **TFRC** ] .  Ref: 12874382 Proc Natl Acad Sci U S AProc Natl Acad Sci U S A, 2003
- At concentrations of  **Tf**  approaching those found in the blood, no differences in  **Tf**  binding to cells were detected, which is consistent with the lower binding constant of HFE for TfR  [ **TFRC** ]  versus  **Tf** .  Ref: 12874382 Proc Natl Acad Sci U S AProc Natl Acad Sci U S A, 2003

**Non-interactive relationship** (e.g. studied together, co-existance, homology, etc.)

- Single value of serum transferrin  [ **TF** ]  receptor  [ **TFRC** ]  is not diagnostic for the absence of iron stores in anaemic patients with rheumatoid arthritis.  Ref: 12755791 Clin Lab Haematol< MedlineTA>Clin Lab Haematol, 2003
- Serum transferrin  [ **TF** ]  receptor  [ **TFRC** ]  sTfR concentrations were measured in anaemic patients with rheumatoid arthritis RA.  Ref: 12755791 Clin Lab Haematol< MedlineTA>Clin Lab Haematol, 2003
- For example, transferrin  [ **TF** ]  receptor  [ **TFRC** ] , a gene known to control cellular iron uptake, was downregulated in the cells treated with the iron containing PA coal in order to protect cells from iron overload.  Ref: 12851122 J Toxicol Environ Health A< MedlineTA>J Toxicol Environ Health A, 2003
- Rottlerin had no effect on cell surface or total expression of the transferrin  [ **TF** ]  receptor  [ **TFRC** ] , providing evidence that the effect on GLAST cannot be attributed to a non specific internalization degradation of plasma membrane proteins.  Ref: 12859677 J Neurochem, 2003
- In contrast, expression of a constitutively active mutant of the Rac1 GTPase inhibits plasma membrane internalization of both the ATP7A and transferrin  [ **TF** ]  receptor  [ **TFRC** ]  transmembrane proteins.  Ref: 12812980 Hum Mol Genet, 2003
- DESIGN AND METHODS Hemoglobin concentration, hematocrit, reticulocyte count, serum ferritin and soluble transferrin  [ **TF** ]  receptor  [ **TFRC** ]  levels were sequentially evaluated in 923 professional football players.  Ref: 12745277 Haematologica, 2003
- RESULTS There were no significant differences in hemoglobin, serum transferrin  [ **TF** ]  receptor  [ **TFRC** ] , copper, or zinc among groups.  Ref: 12885712 Am J Clin Nutr, 2003
- of transferrin  [ **TF** ]  receptor  [ **TFRC** ]  was studied in neuroblastoma derived B103 cells.  Ref: 12833855 Kaibogaku Zasshi, 2003
- Some but not all Ankhzn immunoreactive vesicles simultaneously contained transferrin  [ **TF** ]  receptor  [ **TFRC** ] .  Ref: 12833855 Kaibogaku Zasshi, 2003
- However, cells expressing HFE still showed a decrease in  **Tf**  mediated iron uptake at concentrations of  **Tf**  sufficient to dissociate HFE from the TfR  [ **TFRC** ] .  Ref: 12874382 Proc Natl Acad Sci U S AProc Natl Acad Sci U S A, 2003
- Magnetic activated cell separation studies, using beads to which an anti transferrin  [ **TF** ]  receptor  [ **TFRC** ]  antibody was conjugated, confirmed that in OHSt there was a correspondence between expression of stomatin and the transferrin  [ **TF** ]  receptor  [ **TFRC** ] .  Ref: 12750157 Blood< MedlineTA>Blood, 2003
- Recent reports suggest that the mutations of transferrin  [ **TF** ]  receptor  [ **TFRC** ]  2 TfR2 gene may be involved in non HFE hemochromatosis.  Ref: 12809944 Hepatol Res< MedlineTA>Hepatol Res, 2003
- Surprisingly macrophages exhibit decreased transferrin  [ **TF** ]  receptor  [ **TFRC** ]  concentration in response to inflammatory ligands.  Ref: 12746546 Sci STKE, 2003
- An association was found between the concentration of serum soluble transferrin  [ **TF** ]  receptor  [ **TFRC** ]  and the concentration of Mn in whole blood Pearson s r = 0.48.  Ref: 12884407 J Appl ToxicolJ Appl Toxicol, 2003
- Examples include upregulation of the epidermal growth factor receptor EGFR and the transferrin  [ **TF** ]  receptor  [ **TFRC** ] .  Ref: 12781365 Cancer Cell, 2003
- Ganglioside G M1, a lipid raft marker, and tyrosine phosphorylated proteins, but not CD45 and transferrin  [ **TF** ]  receptor  [ **TFRC** ] , were concentrated in BCR caps, suggesting BCR caps as surface signaling microdomains.  Ref: 12794139 J Immunol, 2003
- Interestingly, our colocalization studies with endosomal markers EEA1, transferrin  [ **TF** ] , and transferrin  [ **TF** ]  receptor  [ **TFRC** ]  suggest that these phospho beta dystroglycan containing internal vesicles represent a subset of recycling endosomes.  Ref: 12795607 Biochemistry< MedlineTA>Biochemistry, 2003
- Expression of dominant negative mutants of the dynamin I, dynamin II and Eps15 proteins that block clathrin dependent endocytosis of the transferrin  [ **TF** ]  receptor  [ **TFRC** ]  do not inhibit internalization of endogenous ATP7A, or an ATP7A reporter molecule CD8 MCF1.  Ref: 12812980 Hum Mol Genet, 2003
- The interface between the two types of regulatory input by insulin is suggested to be the budding of GLUT4 from the transferrin  [ **TF** ]  receptor  [ **TFRC** ]  TfR  [ **TFRC** ]  containing, recycling endosome.  Ref: 12864734 Acta Physiol Scand, 2003
- We further demonstrate that HOCl antagonizes the H2O2 mediated increase in the levels of transferrin  [ **TF** ]  receptor  [ **TFRC** ] , which is a downstream target of IRP1.  Ref: 12888561 J Biol ChemJ Biol Chem, 2003
- , reticulocyte count and soluble transferrin  [ **TF** ]  receptor  [ **TFRC** ] , the 95th percentile of the coefficient of variation distribution was lower than 5% for both hemoglobin and hematocrit.  Ref: 12745277 Haematologica, 2003
- IREs are found in the 5 untranslated region UTR of ferritin Ft and mitochondrial aconitase m Aco mRNAs, and in the 3 UTR of transferrin  [ **TF** ]  receptor  [ **TFRC** ]  TfR  [ **TFRC** ]  and divalent metal transporter 1 DMT1 mRNAs.  Ref: 12855587 Blood< MedlineTA>Blood, 2003
- One is the GFP high fraction containing mainly CFU E and proerythroblasts, which coexpress transferrin  [ **TF** ]  receptor  [ **TFRC** ] , while the other is the GFP low transferrin  [ **TF** ]  receptor  [ **TFRC** ]  negative fraction containing BFU E.  Ref: 12893747 BloodBlood, 2003
- We have previously suggested that phosphatidylinositol 3 kinase PI3K p70 S6 kinase p70 S6K plays an important role in the regulation of neutrophilic differentiation of HL 60 cells on the basis of analysis of transferrin  [ **TF** ]  receptor  [ **TFRC** ]  Trf R positive Trf R and negative Trf R cells that appear after treatment with dimethyl sulfoxide DMSO.  Ref: 12818373 Biochem Pharmacol, 2003
- However, iron status had no significant effect on DMT1 IRE and DMT1 non IRE mRNAs expression in the heart, although it can significantly influence heart transferrin  [ **TF** ]  receptor  [ **TFRC** ]  TfR  [ **TFRC** ]  mRNA expression.  Ref: 12767048 J Cell Physiol, 2003
- Day to day variability in the transferrin  [ **TF** ]  receptor  [ **TFRC** ]  ferritin index in female athletes.  Ref: 12782555 Br J Sports Med, 2003
- Until recently, research results predict that the cellular response to increased NO production should be a decrease in ferritin synthesis, due to IRP1 binding to ferritin mRNA, and an increase in transferrin  [ **TF** ]  receptor  [ **TFRC** ]  biosynthesis, due to IRP1 binding to the transferrin  [ **TF** ]  mRNA.  Ref: 12746546 Sci STKE, 2003
- The measurement of soluble transferrin  [ **TF** ]  receptor  [ **TFRC** ]  sTfR has been proposed as a novel approach to the diagnosis of iron deficiency, especially in anaemia of chronic diseases ACD.  Ref: 12836462 Wien Med Wochenschr, 2003
- Expression of syntaxin 1 alone impaired the cell surface expression of TI VAMP and cellubrevin but not the recycling of transferrin  [ **TF** ]  receptor  [ **TFRC** ] .  Ref: 12759369 J Cell Sci< MedlineTA>J Cell Sci, 2003
- RESULTS Compared with the placebo group, the epoetin alfa treated group responded with increases in hematocrit, reticulocytes, macrocytes, serum EPO, and serum soluble transferrin  [ **TF** ]  receptor  [ **TFRC** ] .  Ref: 12765986 Clin Chem, 2003
- Recombinant human erythropoietin induces a delayed increase in reticulocytosis and in soluble transferrin  [ **TF** ]  receptor  [ **TFRC** ]  levels.  Ref: 12784166 Int J Sports Med, 2003
- CONCLUSION Our studies suggest that expression of wild type HFE in cells produces a decrease in 67 Ga uptake due to a reduction in available  **Tf**  binding sites for 67 Ga  **Tf**  on the TfR  [ **TFRC** ] .  Ref: 12791823 J Nucl Med, 2003
- Replacing the cytoplasmic tail of SR A with that of the transferrin  [ **TF** ]  receptor  [ **TFRC** ]  TfR  [ **TFRC** ]  SR A resulted in retention of the chimeric receptor in the endoplasmic reticulum ER suggesting a specific role for the membrane proximal amino acids in trafficking SR A from the ER to the Golgi.  Ref: 12819208 J Biol Chem< MedlineTA>J Biol Chem, 2003
- Serum transferrin  [ **TF** ]  receptor  [ **TFRC** ]  concentrations were positively correlated with the percentage of hypochromic cells and negatively correlated with MCH.  Ref: 12755791 Clin Lab Haematol< MedlineTA>Clin Lab Haematol, 2003
- High fluorescence reticulocyte counts and soluble transferrin  [ **TF** ]  receptor  [ **TFRC** ]  levels were determined in iron deficiency anemia patients n = 49 and heterozygous beta thalassemia patients n = 43.  Ref: 12870058 Sao Paulo Med J, 2003
- There were no final differences between groups in hemoglobin, plasma ferritin, or transferrin  [ **TF** ]  receptor  [ **TFRC** ]  concentrations or in the ratio of transferrin  [ **TF** ]  receptors to plasma ferritin after control for initial concentrations.  Ref: 12885707 Am J Clin Nutr, 2003
- Genomic DNA was analyzed using primers for beta2 microglobulin and the transferrin  [ **TF** ]  receptor  [ **TFRC** ] .  Ref: 12808124 Lab Invest, 2003
- There was no significant difference in high fluorescence reticulocyte and soluble transferrin  [ **TF** ]  receptor  [ **TFRC** ]  values between the two groups, but a correlation was observed between high fluorescence reticulocytes and soluble transferrin  [ **TF** ]  receptors in iron deficiency anemia, probably due to increased receptor synthesis as a response to decreased iron content in erythrocytes.  Ref: 12870058 Sao Paulo Med J, 2003
- AIM This study compared transferrin  [ **TF** ]  receptor  [ **TFRC** ]  TfR  [ **TFRC** ]  concentrations with iron parameters relative to a child s intake of cow s milk and follow on formula.  Ref: 12856975 Acta Paediatr, 2003
- OBJECTIVE To explore the effect of T 3 on the expression of transferrin  [ **TF** ]  receptor  [ **TFRC** ]  TfR  [ **TFRC** ]  and ferritin Fn in K562 cells and its possible mechanism.  Ref: 12864947 Zhonghua Xue Ye Xue Za Zhi, 2003
- Immunolocalization studies in epithelial cells revealed myo6 was associated with peripherally located vesicles that contained the transferrin  [ **TF** ]  receptor  [ **TFRC** ] .  Ref: 12857860 Mol Biol Cell, 2003
- Soluble transferrin  [ **TF** ]  receptor  [ **TFRC** ]  and immature reticulocytes are not useful for distinguishing iron deficiency anemia from heterozygous beta thalassemia.  Ref: 12870058 Sao Paulo Med J, 2003
- The presence of transferrin  [ **TF** ]  receptor  [ **TFRC** ]  on immature, proliferating thymocytes and the inhibition of thymocyte proliferation and differentiation by anti transferrin  [ **TF** ]  receptor  [ **TFRC** ]  antibody highlight the importance of iron to T cell development.  Ref: 12848962 Autoimmun Rev, 2003
- Cross linking with a stimulatory anti CD28 antibody resulted in an increased expression of CD71  [ **TFRC** ]  transferrin  [ **TF** ]  receptor  [ **TFRC** ]  or CD25 interleukin 2alpha receptor subunit.  Ref: 12853703 Tumour Biol< MedlineTA>Tumour Biol, 2003
- Effect of T 3 on the expression of transferrin  [ **TF** ]  receptor  [ **TFRC** ]  and ferritin in K562 cells and its possible mechanism .  Ref: 12864947 Zhonghua Xue Ye Xue Za Zhi, 2003
- We validated this procedure with two previously characterized cell surface receptors, glutamate receptor subunit A GluR A and the transferrin  [ **TF** ]  receptor  [ **TFRC** ]  TfR  [ **TFRC** ] .  Ref: 12763242 J Neurosci Methods, 2003
- With respect to certain parameters, such as red cell distribution, serum iron binding capacity, transferrin  [ **TF** ]  saturation, transferrin  [ **TF** ]  receptor  [ **TFRC** ] , and transferrin  [ **TF** ]  receptor  [ **TFRC** ]  log ferritin intermittent treatment was superior to the conventional treatment method p < ;.  Ref: 12746164 Pediatr Hematol Oncol, 2003
- Soluble transferrin  [ **TF** ]  receptor  [ **TFRC** ]  and iron status in elderly patients.  Ref: 12836462 Wien Med Wochenschr, 2003
- The regimen outlined in this brief paper focuses on therapeutically utilizing iron and iron compounds in transferrin  [ **TF** ]  receptor  [ **TFRC** ]  rich tumor cells in ways that inhibit and lyse same.  Ref: 12888304 Med Hypotheses, 2003
- In addition, chimeras containing the extracellular domain of the transferrin  [ **TF** ]  receptor  [ **TFRC** ]  and the carboxyl terminal tail of CFTR also enter Rme 1 regulated recycling compartments and accumulates in these compartments containing dominant negative Rme 1, suggesting that in addition to endocytic signals, the carboxyl terminal tail of CFTR also contains intracellular traffic information  Ref: 12839834 Am J Physiol Cell Physiol< MedlineTA>Am J Physiol Cell Physiol, 2003
- Transferrin  [ **TF** ]  receptor  [ **TFRC** ]  1 is differentially required in lymphocyte development.  Ref: 12881306 BloodBlood, 2003
- BACKGROUND Soluble transferrin  [ **TF** ]  receptor  [ **TFRC** ]  sTfR concentration is high in iron deficiency and in conditions of increased erythropoiesis.  Ref: 12867292 Clin Chim ActaClin Chim Acta, 2003
- Although fHFE expression produced an increase in cellular TfR  [ **TFRC** ]  protein, cell surface and intracellular  **Tf**  binding sites were actually decreased in these cells.  Ref: 12791823 J Nucl Med, 2003
- EXPLY 37 R had no effect on T lymphocyte activation, measured as proliferation as well as expression of early and late cell surface antigens CD69, CD25 IL 2R alpha and CD71  [ **TFRC** ]  transferrin  [ **TF** ]  receptor  [ **TFRC** ]  at the cell membrane.  Ref: 12890427 Int Immunopharmacol, 2003
- When fused to unpolarized CD4 or dendritic transferrin  [ **TF** ]  receptor  [ **TFRC** ] , T1 promoted their axonal surface expression.  Ref: 12893943 Science, 2003
- Transferrin  [ **TF** ]  receptor  [ **TFRC** ]  in children and its correlation with iron status and types of milk consumption.  Ref: 12856975 Acta Paediatr, 2003
- Erythroid bone marrow activity and red cell hemoglobinization in iron sufficient b thalassemia heterozygotes as reflected by soluble transferrin  [ **TF** ]  receptor  [ **TFRC** ]  and reticulocyte hemoglobin in content.  Ref: 12801838 Haematologica, 2003
- We have previously shown that mouse embryos homozygous for a disrupted transferrin  [ **TF** ]  receptor  [ **TFRC** ]  allele TfR  [ **TFRC** ]  die of anemia before embryonic day 12.5, although most other embryonic tissues appear to be developing normally.  Ref: 12881306 BloodBlood, 2003
- The geometric mean concentration of soluble transferrin  [ **TF** ]  receptor  [ **TFRC** ]  was lower in the exposed subjects than in the controls 2.2 vs 2.6 mg l 1.  Ref: 12884407 J Appl ToxicolJ Appl Toxicol, 2003
- The response pattern of a possible new marker, beta globin mRNA, is compared with reticulocyte count, levels of haemoglobin, transferrin  [ **TF** ]  receptor  [ **TFRC** ]  and ferritin after r HuEpo treatment.  Ref: 12817911 Scand J Clin Lab Invest, 2003
- Concentrations of hemoglobin, serum ferritin SF, and serum transferrin  [ **TF** ]  receptor  [ **TFRC** ]  TfR  [ **TFRC** ]  were measured at baseline and after 3 and 6 mo.  Ref: 12885710 Am J Clin Nutr, 2003
- GPI 80 positive cells were found in the well differentiated CD11b positive and transferrin  [ **TF** ]  receptor  [ **TFRC** ]  negative cell population.  Ref: 12749849 Exp Cell Res, 2003
- The influence of iron, vitamin B 12, and folate levels on soluble transferrin  [ **TF** ]  receptor  [ **TFRC** ]  concentration in pregnant women.  Ref: 12867292 Clin Chim ActaClin Chim Acta, 2003
- This did not appear to be due to a general effect of the chelators on preventing translation, as the transferrin  [ **TF** ]  receptor  [ **TFRC** ]  1 was markedly up regulated 15 21 fold by DFO or 311.  Ref: 12807743 Carcinogenesis< MedlineTA>Carcinogenesis, 2003
- OBJECTIVE To determine the within subject day to day variability for variables reflecting iron status concentrations of ferritin and soluble transferrin  [ **TF** ]  receptor  [ **TFRC** ]  sTfR, and the sTfR logFerr index in female athletes during a training camp, and in female control subjects.  Ref: 12782555 Br J Sports Med, 2003
